# Effects of chemical fertilizer application on soil properties, banana (*Musa paradisiaca*) yields, and soil microbiomes in a long-term cultivation field

**DOI:** 10.7717/peerj.21912

**Published:** 2026-10-30

**Authors:** Sasipa Pruksangkul, Teerasit Termsaithong, Wuttichai Mhuantong, Tawan Limpiyakorn, Prinpida Sonthiphand

**Affiliations:** 1 Department of Biology, Faculty of Science, Mahidol University, Bangkok, Thailand; 2 Learning Institute, King Mongkut’s University of Technology Thonburi, Bangkok, Thailand; 3 Theoretical and Computational Physics (TCP) group, Center of Excellence in Theoretical and Computational Science (TaCS-CoE), King Mongkut’s University of Technology Thonburi, Bangkok, Thailand; 4 National Center for Genetic Engineering and Biotechnology (BIOTEC), National Science and Technology Development Agency, Pathumthani, Thailand; 5 Department of Environmental Engineering, Faculty of Engineering, Chulalongkorn University, Bangkok, Thailand; 6 Research Unit Control of Emerging Micropollutants in Environment, Chulalongkorn University, Bangkok, Thailand

**Keywords:** Banana yields, Fertilizer, Long-term cultivation field, Soil microbiome

## Abstract

Due to climate change and increasing food demand, conventional agriculture relies heavily on chemical fertilizers. This study investigated the effects of chemical fertilizer application on soil properties, banana (*Musa paradisiaca*) yields, and soil microbiomes in a long-term cultivation field. Metagenomic analysis of the 16S rRNA gene provided a comprehensive overview of soil microbial diversity and functional potential. Nitrogen, phosphorus, and potassium (NPK) fertilizers at 0, 119, 238, and 476 kg ha^−1^ were applied monthly for one year. Fertilizer application significantly altered soil properties and microbial community dynamics but had no significant effect on banana yields. Higher fertilizer rates reduced soil pH and increased available phosphorus and exchangeable potassium, while total nitrogen and organic matter remained relatively unchanged. Elevated NPK inputs increased the abundance of specific microbial taxa, including *Bacillus*, *Streptomyces*, uncultured bacterium JG30-KF-AS9, and *Nitrospira*, while Xanthobacteraceae, bacteriap25, Micromonosporaceae, *Nocardioides*, Subgroup 6, and Gemmatimonadaceae consistently represented core taxa across all treatments. Pearson’s correlation analysis revealed that soil total nitrogen and exchangeable potassium were positively correlated with the abundance of key plant growth-promoting and nitrifying taxa, including *Pseudomonas* and *Nitrospira*, whereas soil pH was negatively correlated with *Acidothermus*, *Nitrospira*, and *Bacillus*, but positively correlated with Subgroup 6. Functional gene profiles further indicated enhanced anaerobic processes associated with carbon, nitrogen, and sulfur cycling. The findings suggest that long-term nutrient accumulation resulting from repeated fertilization and crop residue inputs may sustain banana crops throughout the 10-month production period without additional fertilization. Together, these findings highlight the need for fertilizer regimes tailored to long-term soil nutrient status, contributing to both sustainable agriculture and microbiome-informed crop management.

## Introduction

Bananas (*Musa* spp.) are one of the most important fruit crops globally, widely cultivated across tropical and subtropical regions. They serve as a vital source of nutrition and income for millions of smallholder farmers and play a key role in food and socio-economic security (Lucas & Jomanga, 2021). In Southeast Asia, particularly Thailand, *Musa paradisiaca* is the predominant cultivated species. Bananas are also among the world’s most produced, consumed, and traded agricultural commodities (Bebber, 2023). However, changes in temperature and rainfall resulting from climate change have increasingly affected banana production, threatening economic sustainability in major producing regions (Abdoussalami et al., 2023; Bebber, 2023). To sustain high yields, intensive monoculture systems, chemical fertilizer application, and the cultivation of high-yielding varieties have been widely implemented. In agricultural practice, chemical fertilizers are commonly used to enhance crop production and compensate for nutrient depletion caused by continuous cropping (Abebe et al., 2022). However, excessive or unbalanced fertilizer use can lead to soil degradation, nutrient leaching, and disruption of soil microbiomes, which play key roles in nutrient cycling and are closely associated with plant health (Abebe et al., 2022; Pahalvi et al., 2021). Understanding how fertilizer regimes influence both soil fertility and the soil microbiome is thus essential for developing sustainable banana production systems, particularly in long-term cultivated soils.

Soil microbiomes play vital roles in maintaining soil ecosystem functions by driving nutrient cycling, organic matter decomposition, toxin degradation, and promoting resilience to biotic and abiotic stresses (Ahlawat et al., 2025; Chouyia, Ventorino & Pepe, 2022; Li et al., 2017; Su et al., 2024). They mediate key biogeochemical processes such as nitrification and nutrient mineralization, so sustaining soil fertility and crop productivity. Microbial taxa involved in nitrogen fixation, such as *Bradyrhizobium* and *Rhizobium*, and nitrification, such as *Nitrospira*, have been reported in cultivated and fertilized soils (Kaushal et al., 2025; Wang et al., 2019). In addition, soil microbial taxa such as *Bacillus*, *Pseudomonas*, *Microvirga*, and *Streptomyces* are recognized as plant growth-promoting bacteria due to their ability to facilitate nutrient availability through phosphorus and potassium solubilization, to modulate plant hormones and suppress plant pathogens (Ahlawat et al., 2025; Chen et al., 2026; Kwak et al., 2025; Saxena et al., 2020). Intensive agricultural practices such as excessive fertilization and soil disturbance have degraded soil microbiomes, highlighting the critical need for sustainable management approaches to maintain the microbial stability essential for plant growth (Du et al., 2025; Su et al., 2026). A long-term banana cultivation system provides a unique scenario in which repeated chemical fertilizer inputs and crop residue management may exert cumulative effects on soil health and microbiome composition. Previous studies have examined the effects of various fertilizer regimes on banana productivity and soil properties, primarily under subtropical conditions, with limited integration of soil microbiome analyses (Kumar et al., 2025; Huo et al., 2025; Zhang et al., 2025). However, integrated field-based evidence linking nitrogen, phosphorus, and potassium (NPK) fertilizer regimes to soil properties, microbiome structure, and banana productivity in long-term cultivated tropical soils remains limited.

Monoculture of bananas, particularly in long-term agricultural systems, combined with continuous chemical fertilizer application, may induce distinctive changes in soil properties and microbial communities. However, the linkages between NPK fertilizer application under varying fertilizer regimes, microbial taxa, and banana yields remain poorly understood, especially under tropical field conditions. This study investigated how varying NPK fertilizer regimes affect soil properties, microbiome composition, and *Musa paradisiaca* yields in a long-term tropical cultivation field. We hypothesize that intensive or unbalanced fertilizer application will alter soil physicochemical properties and shift microbial community structure, reducing the abundance of beneficial taxa involved in nutrient cycling and plant growth promotion, and consequently affecting banana productivity. This study therefore integrates soil chemical analyses, amplicon-based microbiome profiling, and yield assessments to elucidate the interrelationships among fertilizer inputs, soil health, and microbial function, providing evidence-based insights for sustainable banana production. This study focused on bulk soil rather than rhizosphere soil, as bulk soil better reflects the overall effects of long-term fertilization on soil properties and microbial community composition. Accordingly, the objectives of this study were to assess the effects of different chemical fertilizer application levels on soil properties, banana yields, and soil microbial communities in a long-term banana cultivation field using field plot experiments, metagenomics, and bioinformatics approaches. Soil microbial functions influenced by chemical fertilizer application were also investigated. This research provides new insights through a comprehensive evaluation of fertilizer-driven interactions among soil properties, crop productivity, and microbial ecology under long-term field-based cultivation conditions. The findings contribute to the development of fertilizer management strategies that optimize banana production while maintaining soil health and microbial balance.

## Materials & Methods

### Sampling sites

The study area has been used for agricultural purposes for 20 years, with plantations of bananas (*Musa paradisiaca*) established within the last 5 years. The land was left for three months before the experiment setup. The study area is located in Nakhon Pathom province, Thailand. Eight experimental plots, each measuring 3  × 70 m, were established, with seven to eight banana trees planted in each plot. Four treatments (*i.e.,* N_0_, N_1/2_, N, and N_2_) were implemented, with each treatment applied to two plots. In this study, NPK fertilizer was applied once a month throughout the 12-month experiment (M1 to M12). The nitrogen, phosphorus, and potassium in the fertilizer were supplied in the forms of ammonium nitrate (NH_4_NO_3_), diphosphorus pentoxide (P_2_O_5_), and dipotassium oxide (K_2_O), respectively. The selected fertilizer application rates were based on the manufacturer’s recommended rate of 238 kg ha^−^^1^, which was used as the standard treatment (N). The other levels were designed to represent a gradient, including a no-fertilizer control (0 kg ha^−^^1^; N_0_), a reduced rate (119 kg ha^−^^1^; N_1/2_), and an elevated rate (476 kg ha^−^^1^; N_2_). The fertilizer was applied evenly around each plot during the first week of every month. All plots were treated consistently throughout the experimental period. Irrigation and herbicide application were conducted monthly, with approximately 1.5 L of water applied per banana tree and glufosinate-ammonium applied at 10 mL/L over 0.16 ha. Soil cover was maintained by recycling banana pseudostems within the plots after harvest, without additional mulch. Soil samples and banana yield data were collected seven days after each fertilizer application. Data were summarized at four time points: T1 (average of M1 to M4), T2 (average of M5 to M8), T3 (average of M9 to M12), and Y1 (average of M1 to M12). Three composite time points were defined by averaging four consecutive monthly measurements: T1, T2, and T3, corresponding to the dry season (December–March), transitional season (April–July), and wet season (August–November), respectively. This classification was based on monthly rainfall patterns recorded throughout the 12-month study period, with rainfall averaged across the same four-month intervals (Fig. S1). This four-month averaging approach reduced short-term month-to-month variation. An annual mean (Y1) was additionally calculated by averaging across all 12 monthly measurements to provide an integrated summary of soil properties and banana productivity under each fertilizer regime over the full experimental year.

### Soil sample collection

Surface soil samples (5–20 cm depth) were collected monthly from December 2021 to November 2022 (M1–M12), resulting in 12 samples per treatment. In total, 48 samples from four treatments (N_0_, N_1/2_, N_1_, and N_2_) were included for soil property and microbiome analyses. Bulk soil was used because it provides a more representative measure of long-term fertilization effects on soil properties and the overall microbial community, independent of root-soil interactions. The soil samples were collected seven days after fertilizer application. For soil analyses, two field plots were established for each treatment, resulting in eight field plots in total (4 treatments × 2 plots). Each month, three soil subsamples were randomly collected from each plot within the banana plantation. Subsamples from the two plots within the same treatment were then pooled to form one composite sample per treatment for laboratory analysis, resulting in 48 monthly composite soil samples over the 12-month experimental period (4 treatments × 12 months). For the seasonal analysis of soil properties, measurements from four consecutive months were averaged to generate one seasonal mean per treatment at each of the three time points (T1, T2, and T3). For microbial community analysis, all 48 monthly composite soil samples were retained to assess temporal dynamics in microbial community composition. The composite samples were separately kept in a plastic bag for soil property analysis. Additionally, the soil samples were stored at −20 °C for DNA extraction.

### Banana yield collection

For each fertilizer treatment, bunch weight (kg), number of hands per bunch (pieces), number of fingers per hand (pieces), finger weight (g), finger diameter (cm), and finger length (cm) were recorded monthly from each field plot. Measurements from the two field plots within the same treatment were averaged to represent the treatment mean for each month. Once the bananas reached their life cycle, they were removed from the plot. Although banana yield data were originally planned to be collected over 12 months, no yields were recorded during the last two months because the production cycle had ended in all plots. Therefore, banana yield data were collected over 10 months (M1–M10) and subsequently summarized at four time points (T1, T2, T3^†^, and Y1^†^). T1, T2, and T3^†^ represent the averages of the first four months, the subsequent four months, and the final two months, respectively. Y1^†^ represents the average yield over the entire 10-month production period.

### Soil property analysis

Total nitrogen (TN) was analyzed by nitrogen combustion according to AOAC International (2019) 993.13. Available phosphorus (AP) was determined using the Bray II method, exchangeable potassium (EK) by flame photometry, organic matter (OM) by the Walkley-Black method, soil pH using a pH meter, and soil texture by mechanical analysis (pipette method) following the Handbook of Soil Analysis (Pansu & Gautheyrou, 2006). All soil parameters were analyzed by the Central Laboratory (Thailand) Co., Ltd. Soil samples were collected monthly over 12 months (M1 to M12) and summarized at four time points (T1, T2, T3, and Y1).

### Soil microbiome analysis

Genomic DNA was extracted using a DNeasy Powersoil Pro Kit (Qiagen). The quality and concentration of the genomic DNA were verified using a NanoDrop spectrophotometer (Thermo Fisher Scientific) and agarose gel electrophoresis. The 16S rRNA gene libraries of all 48 samples were prepared and sequenced on a NovaSeq 6000 according to the manufacturer’s protocol (Novogene Co., Ltd). The V3-V4 region of the 16S rRNA gene was amplified using the forward primer 341F (5′-CCTAYGGGRBGCASCAG-3′) and the reverse primer 806R (5′-GGACTACNNGGGTATCTAAT-3′).

Raw sequencing reads were quality-filtered to remove low-quality sequences (average Phred score < 30) and trimmed to remove barcode and adapter sequences using FASTP (Chen et al., 2018). High-quality reads were filtered, trimmed, and denoised to generate amplicon sequence variants (ASVs) using the DADA2 plugin (Callahan et al., 2016) implemented in QIIME2 (version 2022.2; Bolyen et al., 2019). Taxonomic classification of the representative sequences was performed with the “classify-consensus-blast” method against the SILVA reference database (release 138.1) (Quast et al., 2012) using default parameters. Functional predictions based on 16S rRNA marker gene data were inferred using PICRUSt2 (Douglas et al., 2020). All raw sequencing data were deposited in the NCBI Sequence Read Archive (SRA) under BioProject accession number PRJNA1152543 (Accession SAMN43367503–SAMN43367550).

### Statistical analysis

The effects of fertilizer applications (N_0_, N_1/2_, N, and N_2_) on soil properties and banana yields were analyzed using repeated-measures one-way analysis of variance (ANOVA), and significant differences among treatments were determined by the least significant difference (LSD) using SPSS. A paired *t*-test was used to assess significant differences in the abundance of microbial taxa across fertilizer treatments (*p* ≤ 0.05). Pearson’s correlation coefficients were calculated to examine the relationships among fertilizer treatments, banana yields, soil properties, and microbial taxa (*p* ≤ 0.05) using Microsoft Excel. For analyses linking microbial community composition with soil properties and banana productivity, Pearson’s correlation coefficients were calculated between microbial taxa and soil properties, and between microbial taxa and banana productivity, using all 48 paired monthly observations, with microbial and soil property data matched by treatment and sampling month to ensure direct temporal and treatment-level correspondence. To assess the potential influence of temporal autocorrelation on Pearson’s correlation analyses, the one-month lag autocorrelation of residuals from a linear regression of each genus on each soil property was computed.

## Results

### Effects of fertilizer treatments on soil properties

The effects of fertilizer applications on total nitrogen (TN), available phosphorus (AP), exchangeable potassium (EK), organic matter (OM), and soil pH were investigated (Table 1). Soil texture in all plots was also analyzed at the beginning and end of the experiment and remained classified as clay throughout the cultivation period. The data collected over the year were divided into three time points (T1, T2, and T3), each representing an average of four consecutive months. Additionally, annual data for the entire year (Y1) were also reported. TN content across all treatments (T1, T2, and T3) was comparable and TN levels over the course of the year did not differ significantly among the different NPK fertilizer application rates (Table 1). The varying fertilizer application levels used in this study had no clear impact on TN content in long-term cultivated soil.

**Table 1:** Soil properties under four NPK fertilizer treatments.

		N_0_	N_1/2_	N	N_2_
Total nitrogen (%)	T1	0.18 ± 0.10^a^	0.13 ± 0.05^b^	0.18 ± 0.05^a^	0.18 ± 0.10^ab^
	T2	0.13 ± 0.13^a^	0.13 ± 0.13^a^	0.13 ± 0.13^a^	0.15 ± 0.17^a^
	T3	0.20 ± 0.08^b^	0.25 ± 0.06^a^	0.20 ± 0.08^a^	0.20 ± 0.08^ab^
	Y1	0.17 ± 0.10^a^	0.17 ± 0.10^a^	0.17 ± 0.09^a^	0.18 ± 0.11^a^
Available phosphorus (mg/kg)	T1^*^	453.63 ± 18.32^a^	388.01 ± 80.39^b^	430.73 ± 40.35^ab^	547.20 ± 369.88^ab^
	T2	306.34 ± 45.69^b^	349.08 ± 45.85^a^	347.00 ± 33.06^a^	320.81 ± 45.93^ab^
	T3^*^	307.24 ± 20.77^b^	343.61 ± 41.23^ab^	349.05 ± 26.54^a^	324.60 ± 38.80^ab^
	Y1^*^	355.73 ± 77.50^b^	360.23 ± 56.80^ab^	375.59 ± 50.92^a^	397.53 ± 224.76^ab^
Exchangeable potassium (mg/kg)	T1	480.44 ± 83.72^b^	398.00 ± 112.82^b^	635.92 ± 133.66^a^	545.34 ± 171.89^ab^
	T2	414.71 ± 30.91^b^	531.00 ± 76.26^a^	586.45 ± 56.06^a^	744.88 ± 309.64^a^
	T3	344.38 ± 82.96^a^	398.47 ± 99.92^a^	453.67 ± 41.74^a^	471.41 ± 41.27^a^
	Y1	413.17 ± 86.12^b^	442.49 ± 109.79^b^	558.68 ± 112.53^a^	587.21 ± 221.87^a^
Organic matter (%)	T1	1.08 ± 0.49^a^	1.15 ± 0.41^a^	1.25 ± 0.59^a^	1.31 ± 0.33^a^
	T2^*^	1.97 ± 0.43^b^	2.02 ± 0.43^ab^	2.29 ± 0.38^a^	2.09 ± 0.24^ab^
	T3	2.22 ± 0.11^a^	2.15 ± 0.31^a^	2.29 ± 0.19^a^	2.16 ± 0.25^a^
	Y1^*^	1.75 ± 0.62^b^	1.77 ± 0.58^ab^	1.94 ± 0.64^a^	1.85 ± 0.47^ab^
Soil pH	T1^**^	7.20 ± 0.27^a^	7.41 ± 0.21^a^	6.83 ± 0.39^ab^	6.29 ± 0.55^b^
	T2^**^	7.12 ± 0.20^a^	6.96 ± 0.21^ab^	6.37 ± 0.58^b^	6.15 ± 0.50^c^
	T3^**^	7.32 ± 0.16^a^	6.93 ± 0.55^a^	6.10 ± 0.36^b^	5.97 ± 0.17^b^
	Y1^**^	7.21 ± 0.21^a^	7.10 ± 0.40^a^	6.43 ± 0.52^b^	6.14 ± 0.42^b^

**Notes.**

Significant differences (*p* < 0.10) across treatments are indicated by different letters (a, b, c).

* indicates significance at *p* < 0.05.

** indicates significance at *p* < 0.01.

Although the impact of fertilizer application rates on AP in T1 soils was not clear, AP levels in the N_0_ treatments from the T2 and T3 soils were significantly lower than those in the N treatment (T2: *p* = 0.021, 95% CI [−73.722 to −7.603]; T3: *p* < 0.019, 95% CI [−74.867 to −8.748]) (Table 1). The Y1 data also revealed that AP content in the N_0_ treatment was significantly lower than in the N treatment (*p* = 0.043, 95% CI [−38.945 to −0.772]). Fertilizer application therefore likely increased the amount of AP in this long-term cultivated soil.

The levels of EK in T1 and T3 soils across all fertilizer treatments were comparable; however, those in T2 soils from the N_0_ treatment were significantly lower than in the N_1/2_ (*p* = 0.091, 95% CI [−255.231 to 22.623]), N (*p* = 0.021, 95% CI [−310.379 to −33.116]), and N_2_ (*p* = 0.021, 95% CI [−597.090 to −63.270]) treatments (Table 1). The analysis of Y1 soils showed that EK levels in the N_0_ and N_1/2_ treatments were lower than those in the N and N_2_ treatments (Table 1). Specifically, EK in N_0_ was significantly lower than in N (*p* = 0.003, 95% CI [−225.549 to −65.471]) and N_2_ (*p* = 0.031, 95% CI [−328.139 to −19.938]). EK in N_1/2_ was also significantly lower than in N (*p* = 0.008, 95% CI [−193.123 to −39.259]) and lower than in N_2_ (*p* = 0.064, 95% CI [−300.092 to 10.653]). These results suggest that higher rates of NPK fertilizer application significantly increased EK levels in the cultivated soil.

As with the impact of fertilizer application levels on TN content in soils, OM content across all treatments was relatively comparable, although levels in T2 and Y1 soils from the N treatments were significantly higher than those in the N_0_ treatment (T2: *p* = 0.032, 95% CI [−0.641 to −0.036]; Y1: *p* = 0.031, 95% CI [−0.356 to −0.022]) (Table 1).

Among the soil parameters analyzed in this study, soil pH was the most affected by the fertilizer application level (Table 1). In T1 soils, the N_2_ treatment caused a significant drop in pH within the first three months of cultivation compared to the N_0_ (*p* = 0.003, 95% CI [0.385–1.430]) and N_1/2_ (*p* = 0.002, 95% CI [0.532–1.713]) treatments. A similar decrease in soil pH from the N_1/2_ treatment compared to the N_2_ treatment was also observed in T2 (*p* = 0.013, 95% CI [0.214–1.396]), T3 (*p* = 0.005, 95% CI [0.367–1.548]), and Y1 (*p* <0.001, 95% CI [0.621–1.303]) soils (Table 1). While the N_2_ treatment led to a significant pH decline within the first three months, the N treatment caused a further decline after six months of fertilizer application, indicating that higher NPK application rates accelerated soil acidification. Since pH in T1, T2, T3, and Y1 soils under the N_0_ and N_1/2_ treatments did not differ significantly, the N_1/2_ treatment appears to be suitable for maintaining pH stability and preventing acidification within a year.

### Effects of fertilizer treatments on banana yields

The effects of fertilizer treatments on banana yields, including bunch weight, number of hands per bunch, number of fingers per hand, finger weight, finger diameter, and finger length, were analyzed (Table 2). The banana crops in the N treatment ceased production from M10 to M12, whereas those in the other treatments ceased production from M11 to M12. Therefore, Y1^†^ represents the average banana yield over the 10-month production period. Banana bunch weights in T1 soil under the N_2_ treatment were the highest and were marginally significantly greater than those under the N_0_ treatment. However, bunch weights in T2, T3^†^, and Y1^†^ soils did not differ significantly across the four treatments (Table 2). Other yield parameters, such as number of hands per bunch, number of fingers per hand, finger weight, diameter, and length, did not significantly differ among fertilizer treatments (Table 2). Overall, the results suggest that applying these four fertilizer levels once per month throughout the production period of 10 months did not significantly enhance banana yields under long-term field cultivation.

**Table 2:** Banana yields under four NPK fertilizer treatments.

		N_0_	N_1/2_	N	N_2_
Bunch weight (kg)	T1	11.06 ± 1.69^b^	13.74 ± 2.40^ab^	12.52 ± 1.86^ab^	13.78 ± 1.85^a^
	T2	11.63 ± 1.77^a^	8.98 ± 6.31^a^	10.28 ± 2.70^a^	10.54 ± 2.40^a^
	T3^a^	7.40 ± 1.41^a^	8.10 ± 2.69^a^	4.38 ± 6.19^a^	5.83 ± 0.88^a^
	Y1^a^	10.56 ± 2.25^a^	10.71 ± 4.78^a^	10.00 ± 4.21^a^	10.89 ± 3.55^a^
Number of hands per bunch (pieces)	T1	7.29 ± 0.89^a^	10.43 ± 2.74^a^	9.29 ± 1.99^a^	9.95 ± 2.51^a^
	T2	9.25 ± 3.48^a^	6.18 ± 4.57^a^	7.75 ± 2.87^a^	7.25 ± 0.65^a^
	T3^a^	6.67 ± 0.94^a^	7.00 ± 1.41^a^	4.75 ± 6.72^a^	6.00 ± 0.00^a^
	Y1^a^	7.95 ± 2.39^a^	8.05 ± 3.74^a^	7.76 ± 3.48^a^	8.08 ± 2.25^a^
Number of fingers per hand (pieces)	T1	10.97 ± 2.33^a^	12.15 ± 1.54^a^	12.08 ± 0.59^a^	13.00 ± 2.08^a^
	T2	13.60 ± 1.40^a^	10.95 ± 7.35^a^	13.40 ± 0.85^a^	12.45 ± 0.87^a^
	T3^a^	12.90 ± 0.42^ab^	14.90 ± 0.42^a^	7.80 ± 11.03^b^	14.50 ± 1.84^a^
	Y1^a^	12.41 ± 2.02^a^	12.22 ± 4.60^a^	11.75 ± 4.31^a^	13.08 ± 1.64^a^
Finger weights (g)	T1	104.33 ± 1.15^a^	103.50 ± 3.42^a^	104.50 ± 6.61^a^	103.25 ± 2.50^a^
	T2	99.00 ± 6.00^a^	78.00 ± 52.03^a^	99.25 ± 3.59^a^	96.50 ± 1.91^a^
	T3^a^	94.00 ± 1.41^a^	94.50 ± 4.95^a^	49.50 ± 70.00^b^	93.50 ± 2.12^a^
	Y1^a^	100.13 ± 5.43^a^	91.50 ± 32.49^a^	91.40 ± 32.51^a^	98.60 ± 4.60^a^
Finger diameters (cm)	T1	3.34 ± 0.27^a^	3.39 ± 0.28^a^	3.33 ± 0.32^a^	3.53 ± 0.45^a^
	T2	3.70 ± 0.40^a^	2.82 ± 1.89^a^	3.99 ± 0.19^a^	3.72 ± 0.19^a^
	T3^a^	3.32 ± 0.02^a^	3.25 ± 0.14^ab^	1.83 ± 2.58^b^	3.28 ± 0.15^a^
	Y1^a^	3.48 ± 0.34^a^	3.13 ± 1.14^a^	3.29 ± 1.22^a^	3.56 ± 0.33^a^
Finger lengths (cm)	T1	11.24 ± 1.38^a^	11.96 ± 1.31^a^	11.76 ± 0.45^a^	11.34 ± 1.31^a^
	T2	11.46 ± 1.83^a^	9.12 ± 6.23^b^	11.91 ± 1.54^a^	10.56 ± 2.43^b^
	T3^a^	8.95 ± 5.17^ab^	9.21 ± 5.32^a^	4.57 ± 4.57^b^	8.45 ± 4.89^ab^
	Y1^a^	10.87 ± 1.67^a^	10.27 ± 3.96^a^	10.38 ± 3.86^a^	10.45 ± 1.96^a^

**Notes.**

Significant differences (*p* < 0.10) across treatments are indicated by different letters (a, b, c).

T1^a^, T2^a^, and T3^a^ indicate mean yields for months M1-M4, M5-M8, and M9-M10, respectively; Y1^a^ indicates the mean yield for M1-M10.

### Soil microbiome dynamics under varying NPK fertilizer

The soil microbiome in a total of 48 samples, collected from four fertilizer treatments (N_0_, N_1/2_, N, and N_2_) with 12 samples per treatment, was analyzed by the analysis of the V3-V4 region of the 16S rRNA gene. After removing chimeras and low-quality sequences, a total of 4,224,443 sequences were obtained, with a read depth ranging from 65,800 to 107,709 sequences per sample. The average sequence length was 409 bp. Across all samples, 6,977 ASVs were detected after rarefying the unequal sequencing depths to 42,446 sequences per sample.

The relative abundance of soil microbiomes at the phylum level was comparable across all analyzed samples and treatments (Fig. 1). The major phyla detected were Proteobacteria (18–57%), Actinobacteria (12–40%), Chloroflexi (4–24%), Acidobacteria (6–15%), Firmicutes (3–19%), and Gemmatimonadetes (1–6%). Minor detected phyla included Bacteroidetes, Verrucomicrobia, Cyanobacteria, Nitrospirae, Rokubacteria, and Latescibacteria (Fig. 1).

To gain insight into microbial shifts, soil microbiomes were analyzed at the level of the genus (Fig. 2). The relative abundance of uncultured Roseiflexaceae, members of Chloroflexi, was consistently high (average 7–9%) in all soil samples, although it slightly decreased with increasing fertilizer application. The relative abundances of *Bacillus* (average 7%), *Streptomyces* (average 4%), uncultured bacterium JG30-KF-AS9 (average 2%), and *Nitrospira* (average 3%) in the N_2_ treatment were significantly higher than those in the other three treatments (averages of 4–6%, 3–4%, 0–1%, and 1–2%, respectively) (*p* < 0.05). The results suggested that *Bacillus*, *Streptomyces*, uncultured bacterium JG30-KF-AS9, and *Nitrospira* were significantly enhanced by the N_2_ treatment, the highest amount of fertilizer application.

**Figure 1:**
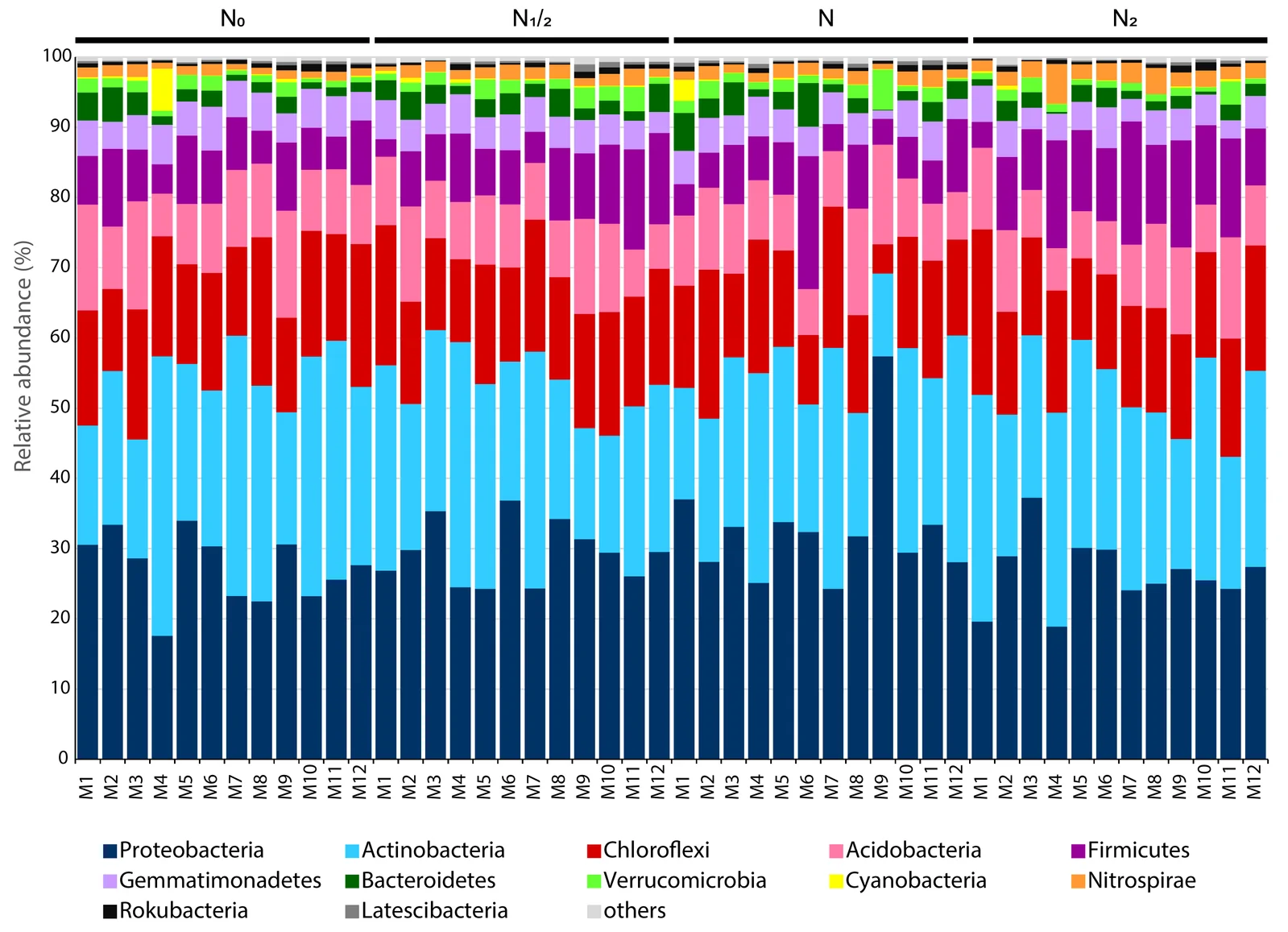
Relative abundance of microbial communities at the phylum level under four fertilizer treatments (N_0_, N_1/2_, N, and N_2_) over a 12-month period (M1 to M12). The corresponding fertilizer application rates were 0, 119, 238, and 476 kg ha^−1^, respectively.

**Figure 2:**
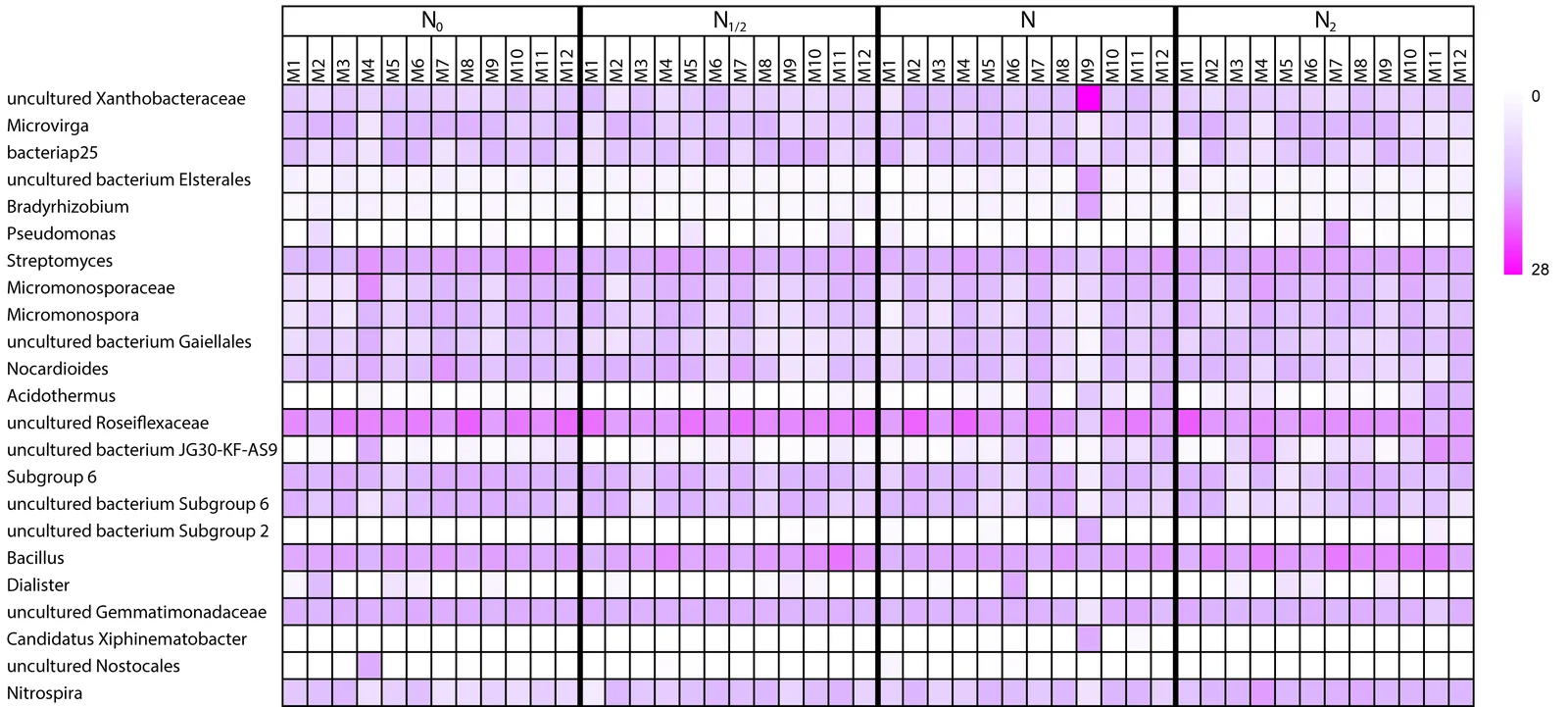
Heatmap showing the relative abundance of microbial communities at the genus level under four fertilizer treatments (N_0_, N_1/2_, N, and N_2_) over a 12-month period (M1 to M12). The corresponding fertilizer application rates were 0, 119, 238, and 476 kg ha^−1^, respectively.

Other dominant taxa that were consistently present across all fertilizer treatments included uncultured Xanthobacteraceae (average 1–4%), bacteriap25 (average 1–2%), Micromonosporaceae (average 2%), *Nocardioides* (average 2%), Subgroup 6 (average 2–3%), and uncultured Gemmatimonadaceae (average 2–3%) (Fig. 2). These taxa did not appear to be influenced by the four differing fertilizer application rates.

### Relationship between soil properties and soil microbial taxa

Multivariate analyses canonical correspondence analysis (CCA) and redundancy analysis (RDA), showed no statistically significant relationships between soil properties and microbial communities. In this study, bivariate analysis was considered more appropriate than multivariate analysis, as it provided a clearer understanding of the linear relationships between specific pairs of variables. Moreover, bivariate analysis also helped identify variables that were non-significant or unassociated.

Pearson’s correlation coefficient revealed a significant positive correlation between TN and the abundance of both *Pseudomonas* (*r* = 0.31, *p* < 0.05) and uncultured bacterium *Gaiellales* (*r* = 0.32, *p* < 0.05) (Table S1). EK was significantly positively correlated with the abundance of *Pseudomonas* (*r* = 0.56, *p* < 0.05) and *Nitrospira* (*r* = 0.54, *p* < 0.05). Soil pH was significantly positively correlated with uncultured bacterium Subgroup 6 (*r* = 0.44, *p* < 0.05), but significantly negatively correlated with uncultured bacterium *Gaiellales* (*r* = −0.41, *p* < 0.05), *Acidothermus* (*r* = −0.58, *p* < 0.05), uncultured bacterium JG30-KF-AS9 (*r* = −0.54, *p* < 0.05), *Bacillus* (*r* = −0.40, *p* < 0.05), and *Nitrospira* (*r* = −0.54, *p* < 0.05). AP and OM showed no significant correlation with the abundance of any microbial genera (Table S1). The relationships between banana yields and soil microbial taxa over the 10-month production period were also analyzed using Pearson’s correlation; however, no significant relationships were identified (Table S1).

### Microbial functions in long-term cultivated soils

Genes related to the carbon, nitrogen, and sulfur cycles were extracted from the KEGG database to infer soil microbial functions driving key nutrient cycles. Among all retrieved genes, those associated with these three cycles were detected at low levels, accounting for only ∼1% in each sample (Fig. 3). Although genes involved in methane oxidation were detected, most carbon-cycle genes were related to methanogenesis, with substrates CO_2_ (0.04–0.08%), acetate (0.15–0.19%), methanol (0.03–0.05%), and methylated amines (0.04–0.07%) (Fig. 3).

**Figure 3:**
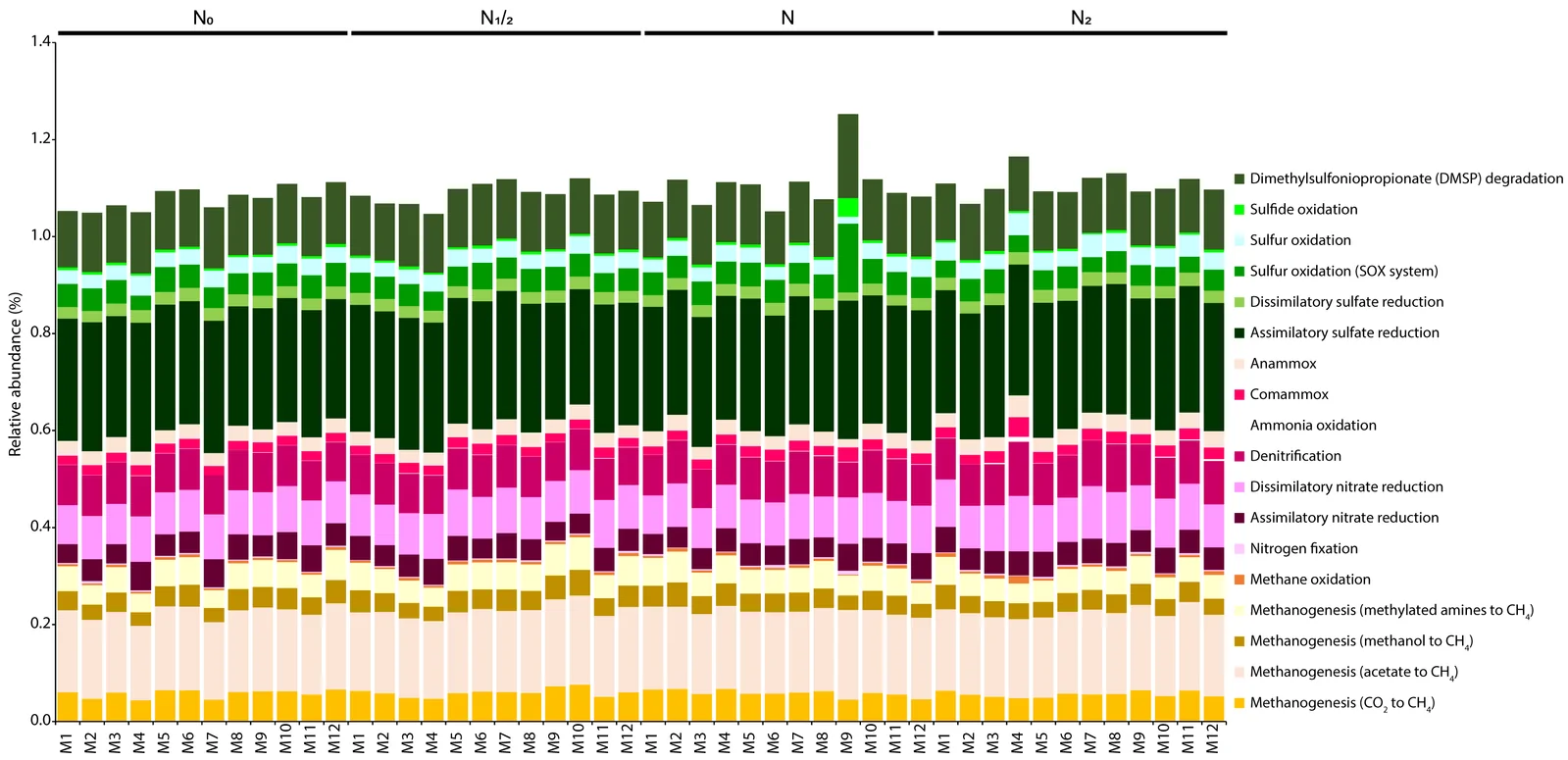
Relative abundance of functional genes associated with carbon, nitrogen, and sulfur cycling under four fertilizer treatments (N_0_, N_1/2_, N, and N_2_) over a 12-month period (M1 to M12), based on KEGG annotations. The corresponding fertilizer application rates were 0, 119, 238, and 476 kg ha^−1^, respectively.

Genes associated with multiple nitrogen-cycle processes were also identified; however, most were linked to anaerobic pathways. The dominant anaerobic nitrogen-cycle genes were those for dissimilatory nitrate reduction (0.08–0.11%), denitrification (0.07–0.11%), assimilatory nitrate reduction (0.04–0.06%), and anammox (0.02–0.04%) (Fig. 3). Other nitrogen-related functional genes were comammox (0.02–0.04%), ammonia oxidation (≤0.01%), and nitrogen fixation (≤0.01%).

For the sulfur cycle, the most abundant genes across all samples were those involved in assimilatory sulfate reduction (0.24–0.29%) and dimethylsulfoniopropionate (DMSP) degradation (0.11–0.17%) (Fig. 3). Genes associated with sulfur oxidation *via* the SOX system (0.03–0.14%) and other sulfur oxidation pathways (0.01–0.05%) were also detected. Genes involved in dissimilatory sulfate reduction were present at low proportions (0.02–0.03%), and genes associated with sulfide oxidation were distinctly abundant in one sample (0.04%) but occurred at low levels in the others (Fig. 3).

## Discussion

### Soil acidification induced by fertilizer application without corresponding increases in banana yields

Among soil properties, pH was clearly and significantly affected by the level of fertilizer application (Table 1). Applying fertilizer at or above 238 kg ha^−^^1^ once a month for a year enhanced soil acidification. In our study, the pH values influenced by fertilizer treatments remained within the optimal range for cultivation (Singh et al., 1995); however, potential acidification was observed at higher fertilizer rates. Nitrogen fertilization exceeding 200 kg ha^−^^1^ yr^−^^1^ promoted acidification in surface farmland soils (Li et al., 2019). Since ammonium nitrate (NH_4_NO_3_) was the nitrogen source used in this study, it enhanced nitrification, leading to proton (H^+^) release and a consequent decrease in soil pH. The decline in soil pH was strongly associated with nitrification potential and net proton release, indicating that high-rate ammonium nitrate fertilization accelerated soil acidification (Cai et al., 2014; Tkaczyk et al., 2020). Additionally, a global meta-analysis reported that urea and ammonium nitrate contributed more to soil acidification than ammonium-based fertilizers (Tian & Niu, 2015).

Higher application rates of NPK fertilizer resulted in greater amounts of AP and EK in the long-term cultivation soils, suggesting that phosphorus and potassium were either limiting nutrients or that fertilizer applications exceeding 119 kg ha^−^^1^ surpassed the nutrient uptake capacity of bananas (Table 1). A global meta-analysis revealed that crop production is commonly co-limited by N, P, and K (Fang et al., 2024). However, NPK fertilization did not increase banana yields in the long-term cultivation land (Table 2), indicating that background soil nutrient levels were already optimal for banana growth.

Although phosphorus and potassium are essential macronutrients required in large quantities by crops, both remain largely immobile in soil because they are retained within the solid fraction (Delgado et al., 2024). Phosphate has limited mobility as it adsorbs onto clay particles, iron oxides, and hydroxides (Huo, Min & Wang, 2021). This likely contributed to the consistent phosphorus content observed in the clay-textured fertilized soils throughout the year. Potassium dynamics in soil are influenced by clay quality, pH, and cation exchange capacity (Gurav et al., 2024).

Fertilizer applications at 0, 119, 238, and 476 kg ha^−^^1^ had no clear impact on TN and OM contents or on banana yields in the long-term cultivated soil (Tables 1 and 2), indicating nutrient saturation, high background fertility, organic matter decomposition, soil texture and nitrogen losses. Long-term cultivation with chemical fertilizer application may have already resulted in sufficient nutrient levels in the soil. Metadata analysis revealed that applying synthetic nitrogen fertilizer on farms globally substantially slowed down the loss of OM from agricultural soils (Ladha et al., 2011). Previous studies suggested that nitrogen enhances OM stabilization by modifying the metabolic capacity of soil microbiomes and reducing overall microbial activity, leading to greater stability of soil OM accumulation (Li et al., 2023; Zhou et al., 2014). Nutrient cycling processes such as mineralization, immobilization, and losses may already have reached equilibrium, so that additional fertilizers applied neither accumulate further in the soil nor enhance crop yield. The balance between inputs (fertilizers and crop residues) and outputs (crop uptake and losses) may have reached a steady state, leading to relatively constant TN and OM contents despite variations in fertilizer rate.

Another possible explanation is high soil background fertility. Since the land in our study area has been used as agricultural land for long time, initial OM and TN contents were moderate to high even though the land was left for three months before the experiment commenced. Additional fertilizer inputs may not significantly alter total contents. Long-term cultivated soils tend to retain nitrogen fertilizer through incorporation into OM or adsorption onto colloids, buffering soil nitrogen availability over time (Li et al., 2023; Sebilo et al., 2013).

Organic matter decomposition may also have contributed to the stable TN and OM levels in soils under fertilizer application. Continuous cultivation and fertilization can stimulate microbial activity, accelerating OM decomposition (Li et al., 2020). This decomposition process can balance TN and OM inputs from plant residues. The decrease in soil pH observed in this study could reduce OM mineralization by inhibiting microbial activity (Li et al., 2023). This can contribute to the retention of OM in the soil over time.

Clay- and silt-rich soils support the storage of nutrients, including carbon and nitrogen (Kuśmierz et al., 2023; Matus, 2021). This can buffer the effects of different fertilizer applications on TN and OM levels. Moreover, clay particles generally reduce the mobility of OM and TN within the soil by promoting their stabilization and retention (Ding et al., 2021). This could also delay nutrient mobility to bananas.

After application, ammonium nitrate fertilizer dissolves in water, releasing ammonium (NH_4_^+^) and nitrate (NO_3_^−^) ions. This stimulated nitrification driven by ammonia-oxidizing (AOB) and nitrite-oxidizing bacteria (NOB), respectively. Nitrate ions (NO_3_^−^) derived from both the dissolved fertilizer and nitrification can either be taken up by plants, lost through denitrification, or leached into the environment. Members of microbial taxa such as Nitrospirae, and Proteobacteria, which are involved in nitrification and denitrification (Li et al., 2017; Su et al., 2024; Wang et al., 2019), were detected in the analyzed soils. Consequently, the applied fertilizers may not have significantly affected TN levels or banana yields, possibly due to nitrate losses through leaching and denitrification, particularly under the clay-textured soil conditions. It should be noted that the stabilization mechanisms of TN and OM in the fields are complex and involve a combination of adsorption, co-precipitation, retention, redox processes, and microbial decomposition and activities (Li et al., 2023).

In addition, the genetics of banana plants could also limit both the quality and quantity of yield. Fruit length, number of hands per bunch, and number of fruits per bunch are determined mainly by genetic traits rather than by fertilizer application (Nomura et al., 2017). Bananas with different genetic backgrounds also vary in their tolerance to abiotic stresses, resulting in differential growth and yield responses under limiting conditions (Abdoussalami et al., 2023; Rapetti et al., 2025). Beyond genetics, climate conditions and cultivation practices further modulate banana yield and quality (Abdoussalami et al., 2023; Nofal & Rezk, 2021). A systematic review showed that banana production is sensitive to environmental variation, including elevation, geographic location, drought, water stress, and temperature (Abdoussalami et al., 2023). In our study area, annual rainfall was less than 1,000 mm, which is below the 1,100 mm threshold associated with significant yield losses in East African highland banana cultivation (Van Asten, Fermont & Taulya, 2011), while the annual average temperature of 28.2 °C fell within the optimal range of 20−35 °C for banana growth (Bahadur, Anmol & Singh, 2020). The planting distance of 3  × 70 m with seven to eight banana plants per plot may be suitable for banana cultivation in this study, as yields were relatively comparable across fertilizer levels. Previous studies suggested that macronutrients, especially phosphate, nitrogen, and potassium, are essential for enhancing banana yields (Kumar & Pandey, 2008; Nofal & Rezk, 2021). Since the background soil in our study was already nitrogen-saturated, a fertilizer ratio with higher phosphate and potassium content may be more effective in improving banana productivity.

### Alterations in soil microbiomes in response to fertilizer application

Key microbial phyla (*e.g.*, Proteobacteria, Actinobacteria, Chloroflexi, Acidobacteria, and Firmicutes) in background and fertilized banana cultivated soils were comparable to those in other banana-associated soils (Kaushal et al., 2025; Yao et al., 2024). The abundance of Actinobacteria and Chloroflexi, which are filamentous bacterial species, increased with fine-soil textures (Xia, Rufty & Shi, 2020). Their high abundance in our study may be attributed to the clay soil texture. Uncultured Roseiflexaceae, members of Chloroflexi, were dominant in all soil conditions (Fig. 2). These microbial taxa were identified as bacterial biomarkers of bulk soil from tobacco fields; were also commonly present in the surface layers of nutrient-rich aquatic environments; and are involved in carbon dioxide assimilation (Deng et al., 2024; Shi et al., 2020). An association of Roseiflexaceae with soil ammonia oxidation was also reported (Yi et al., 2022).

Among the detected genera, Xanthobacteraceae, bacteriap25, Micromonosporaceae, *Nocardioides*, Subgroup 6, and uncultured Gemmatimonadaceae could represent the core soil microbiome because they were consistently present in all soil conditions (Fig. 2). Xanthobacteraceae are a well-known family of nitrogen-fixing bacteria and are also capable of oxidizing nitrite to nitrate, considered NOB (González-Cortés et al., 2022; Su et al., 2024). The consistent abundance of Xanthobacteraceae may have contributed to maintaining TN levels in our study plot. Genome analysis of Micromonosporaceae revealed genes associated with stress responses and plant interactions, indicating their adaptive versatility and potential agricultural benefits (Carro et al., 2018). *Nocardioides*, commonly found in soils including those treated with NPK fertilizer, play key roles in degrading organic pollutants and may have contributed to maintaining OM levels in the study area (Huq et al., 2024; Francioli et al., 2016; Yoon & Park, 2006). Gemmatimonadaceae are abundant in soil, frequently found in the rhizosphere, and their presence was linked to the strengthening of the nitrification function in rhizosphere soil in slightly Cd-contaminated farmland (Liu et al., 2022; Mujakić, Piwosz & Koblížek, 2022). While proteobacterial bacteriap25 and acidobacterial Subgroup 6 have been observed in other soils (Dong et al., 2024), their ecological roles are not well documented. Overall, core microbial taxa could play a role in maintaining nitrogen and carbon in long-term cultivated and fertilized soils.

Fertilizer application at 476 kg ha^−^^1^ once per month for one year enhanced the abundance of *Bacillus*, *Streptomyces*, uncultured bacterium JG30-KF-AS9, and *Nitrospira*. Although uncultured bacterium JG30-KF-AS9 has rarely been reported in soil environments, the beneficial genera *Bacillus*, *Streptomyces*, and *Nitrospira* are commonly found in banana-associated soils (Kaushal et al., 2025; Muzami et al., 2025; Yao et al., 2024). The increase in the relative abundance of *Bacillus*, *Streptomyces*, uncultured bacterium JG30-KF-AS9, and *Nitrospira* in banana-cultivated soils following NPK fertilizer applications can be attributed to their ecological and metabolic adaptation to nutrient-enriched environments. NPK fertilizers can alter soil nutrient dynamics, pH, and microbial resource availability, creating favorable conditions for them.

*Bacillus* are ubiquitous in soils and occur across a wide range of ecological habitats, including nutrient-rich environments (Francioli et al., 2016; Saxena et al., 2020). They exhibit versatile metabolic capabilities, including nitrogen fixation and the solubilization of phosphorus and potassium, and are known as plant growth-promoting bacteria (Ahlawat et al., 2025; Saxena et al., 2020). These characteristics contribute to both soil health and crop productivity.

A previous study also reported that long-term fertilizer application increased the relative abundance of *Streptomyces* in soils (Francioli et al., 2016). As in *Bacillus*, *Streptomyces* can solubilize phosphorus, supporting plant growth (Ahlawat et al., 2025; Chouyia, Ventorino & Pepe, 2022). Additional contributions of *Streptomyces* to sustaining soil fertility and crop production include their plant growth-promoting ability and their production of phytohormones, bioactive compounds, metabolites, and antibiotics (Chouyia, Ventorino & Pepe, 2022; Francioli et al., 2016).

*Nitrospira*, key NOB, convert nitrite to nitrate, a major nitrogen source for plants. Higher nitrogen input from NPK fertilizers increases nitrite production by ammonia oxidizers, stimulating *Nitrospira* growth. In addition, complete ammonia-oxidizing (comammox) *Nitrospira*, which convert ammonia directly to nitrate, have also been identified and found to increase in abundance in long-term fertilized soils (Wang et al., 2019). However, to clarify their niche partitioning and nitrification activity across different fertilizer application levels, direct measurements of nitrification activities should be conducted. The rising abundance of both NOB *Nitrospira* and comammox *Nitrospira* under high fertilizer application may either enhance banana production or contribute to nitrogen loss through leaching. Evidence also suggests that NPK fertilizers can enhance beneficial soil microbes, specifically those that fix nitrogen and solubilize phosphorus for plants (Verma et al., 2023).

Taking into account biogeographical and abiotic factors, pH was identified as the key factor controlling soil microbiomes globally (Zhou et al., 2024). In this study, among soil properties, soil pH was most strongly affected by NPK fertilizer levels, which subsequently influenced soil microbial taxa. As soil pH decreased, the relative abundance of uncultured bacterium *Gaiellales*, *Acidothermus*, uncultured bacterium JG30-KF-AS9, and *Nitrospira* significantly increased. Both *Gaiellales* and *Acidothermus* showed greater adaptability to acidic soils (Liang et al., 2021; Zhou et al., 2024). Although JG30-KF-AS9 have previously been detected in agricultural soils, reports on their association with soil pH remain limited. Both NOB and comammox *Nitrospira* have been found active in and isolated from acidic soils (Takahashi et al., 2025). In contrast, the significant positive correlations between soil pH and the relative abundance of uncultured bacterium Subgroup 6 suggested that this taxon may be less adapted to acidic soils.

The significant positive correlations between EK and the relative abundance of *Pseudomonas* and *Nitrospira* suggested that potassium availability enhanced their metabolic activity and growth. *Pseudomonas* are widely recognized as plant growth-promoting bacteria capable of solubilizing nutrients (Ahlawat et al., 2025; Li et al., 2017). Their increased abundance under higher EK could reflect improved nutrient exchange in soils, possibly contributing to extra availability of EK in the NPK fertilized soils. Similarly, the positive correlation between EK and *Nitrospira* suggests that potassium availability may indirectly favor nitrifying activity. Collectively, our results suggest that *Nitrospira* abundance was significantly associated with higher fertilizer levels, greater potassium availability, and lower pH. This aligns with previous studies demonstrating that fertilizer application drives soil acidification and potassium enrichment, both of which are key factors shaping *Nitrospira* abundance in agricultural soils (Han, Huang & Chen, 2021; Lin et al., 2022).

The results also suggested that high TN levels were positive significantly associated with the abundance of *Pseudomonas* and uncultured bacterium *Gaiellales* (Table S1). *Pseudomonas* species capable of nitrogen fixation have been reported in other cultivated soils and could be responsible for elevated TN (Li et al., 2017; Singh et al., 2024). In addition, long-term chemical fertilizer application has been shown to increase the abundance of *Gaiellales* in Larix-associated soils (Wang et al., 2024).

Overall, soil microbial functions driving key nutrient cycles over the one-year cultivation period were consistent across the four treatments and were dominated by anaerobic processes, including methanogenesis, denitrification, and sulfate reduction (Fig. 3). These anaerobic processes may be enhanced by the clay soil texture. A previous study suggested that, after pH, soil texture was the next most important factor shaping soil microbiome structure (Xia, Rufty & Shi, 2020). Genes involved in methanogenesis, an anaerobic pathway for organic matter degradation, were highly abundant compared with other carbon-cycle genes (Fig. 3). The relative abundances of genes responsible for organic compound degradation increase in fine-textured soils (Xia, Rufty & Shi, 2020). This anoxic microenvironment created by clay particles may be an important factor controlling the final steps of carbon decomposition. Two major nitrogen-cycle processes detected in this study were dissimilatory nitrate reduction and denitrification. In rice paddies receiving high nitrogen fertilization over long periods, denitrification is typically the main nitrate-reducing process (Pandey et al., 2019). However, as soils become more anoxic, nitrate consumption shifts from denitrification toward dissimilatory nitrate reduction to ammonia (DNRA) (Friedl et al., 2018). Thus, soil texture and fertilizer application in this study likely influenced the dynamics of nitrogen-cycle genes. The dominance of assimilatory sulfate reduction and DMSP degradation genes suggests that the sulfur cycle is primarily driven by microbial biomass production and plant organic matter interactions rather than anaerobic respiration. Sulfur tends to be more abundant in fine-textured soils, including clay-rich soils, largely due to their higher OM content (Chaudhary et al., 2023). However, functional profiles predicted using PICRUSt2 represent potential metabolic capabilities inferred from taxonomic composition. Therefore, validation using metatranscriptomics, enzyme assays, or direct measurements of microbial activity is needed to confirm actual soil microbial functions.

## Conclusions

Four levels of chemical fertilizer application (0, 119, 238, and 476 kg ha^−^^1^, corresponding to N_0_, N_1_/_2_, N, and N_2_, respectively) were applied monthly for one year in a long-term banana cultivation field. Soil properties, banana yields, and soil microbiomes influenced by NPK fertilizers were analyzed. Fertilizer application significantly affected soil properties and microbial community dynamics but did not significantly influence banana yields, leading to consistent productivity. Higher NPK levels significantly lowered soil pH and increased AP and EK, while TN and OM remained comparable. Elevated fertilizer levels also increased the relative abundance of *Bacillus*, *Streptomyces*, uncultured bacterium JG30-KF-AS9, and *Nitrospira*, while the core soil microbial taxa identified were Xanthobacteraceae, bacteriap25, Micromonosporaceae, *Nocardioides*, Subgroup 6, and uncultured Gemmatimonadaceae. These increased and core taxa are involved in nutrient cycling, organic matter decomposition, and plant growth promotion through phosphorus and potassium solubilization. The positive associations between TN and EK with the abundance of plant growth-promoting and nitrifying taxa, including *Pseudomonas* and *Nitrospira*, further underscore the role of nutrient availability in shaping beneficial microbial communities. Conversely, soil pH exerted differential effects on microbial taxa, positively associated with Subgroup 6, but negatively associated with *Acidothermus*, *Nitrospira*, and *Bacillus*, highlighting soil pH as an important property linked to microbial community composition under long-term fertilization. The functional genes retrieved support anaerobic processes that drive the carbon, nitrogen, and sulfur cycles. This study suggests that nutrient accumulation resulting from long-term fertilizer application and crop residue inputs in soil cultivated for over 20 years may be sufficient to sustain banana growth during the 10-month cultivation period without additional fertilization. From a practical perspective, no statistically significant differences in banana yields were observed across fertilizer treatments, suggesting that increasing fertilizer rates may not confer measurable yield advantages under the long-term cultivation and smallholder practices (*e.g.*, monthly irrigation and herbicide application, soil cover with banana pseudostems, and seven to eight banana trees per 3  × 70 m plot). Therefore, emphasis should be placed on site-specific management. Achieving higher banana yields while maintaining soil health requires appropriate agricultural practices that consider fertilizer type, application rate, and existing soil nutrient status. Therefore, optimal fertilizer rates may vary depending on specific field conditions. Since this study focused mainly on bulk soils, including infertile and rhizosphere soils in future analyses would provide a more comprehensive understanding of fertilizer impacts on soil microbial dynamics and plant-microbe interactions. Additionally, increasing the number of plots per treatment beyond two would strengthen the robustness of the statistical analysis.

##  Supplemental Information

10.7717/peerj.21912/supp-1 Supplemental Information 1 Monthly and average rainfall Monthly rainfall recorded during the 12-month experimental period (A), and average rainfall for the four-month intervals corresponding to the dry season (T1), transitional season (T2), and wet season (T3), along with the annual average (Y1) (B). Rainfall data were obtained from the Thai Meteorological Department https://www.tmd.go.th/

10.7717/peerj.21912/supp-2 Supplemental Information 2 Pearson’s correlations of microbial genera with soil properties and with banana yields

10.7717/peerj.21912/supp-3 Supplemental Information 3 Raw data

## Additional Information and Declarations

### Data Availability

The following information was supplied regarding data availability: The data is available in the Supplemental Files. The raw sequence data are available at NCBI BioProject: PRJNA1152543 (SAMN43367503–SAMN43367550).
