# Supplementary material for: Effects of chemical fertilizer application on soil properties, banana (Musa paradisiaca) yields, and soil microbiomes in a long-term cultivation field
Source: PeerJ. 2026 Oct 30;14:e21912. doi: 10.7717/peerj.21912 (PMC13726574; doi:10.7717/peerj.21912)
Supplement: Supplementary file 1 [file peerj-14-21912-s001.pdf]

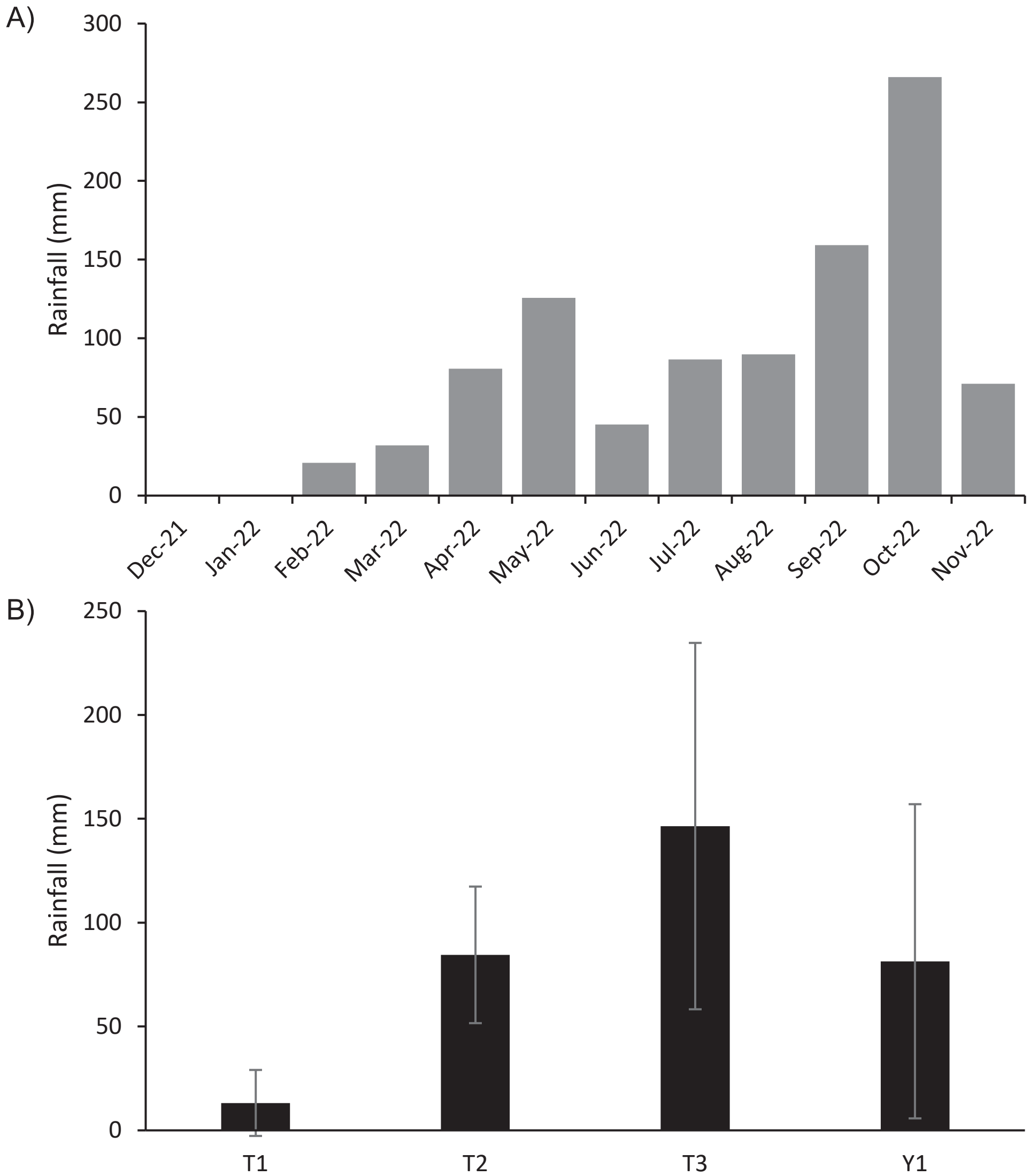
A)
Rainfall (mm)
300
250
200
150
100
50
0
Dec-21
Jan-22
Feb-22
Mar-22
Apr-22
May-22
Jun-22
Jul-22
Aug-22
Sep-22
Oct-22
Nov-22
B)
Rainfall (mm)
250
200
150
100
50
0
T1
T2
T3
Y1
