# Supplementary material for: Effects of chemical fertilizer application on soil properties, banana (Musa paradisiaca) yields, and soil microbiomes in a long-term cultivation field
Source: PeerJ. 2026 Oct 30;14:e21912. doi: 10.7717/peerj.21912 (PMC13726574; doi:10.7717/peerj.21912)
Supplement: Supplementary file 2 [file peerj-14-21912-s002.docx]

Table S1. Pearson’s correlations of microbial genera with soil properties and with banana yields.

| Genus | Soil properties | | | | | Banana yields | | | | | |
| --- | --- | --- | --- | --- | --- | --- | --- | --- | --- | --- | --- |
|  | TN | AP | EK | OM | pH | Bunch Weight | Hand per Bunch | Finger per Hand | Finger Weight | Finger Diameter | Finger Length |
| uncultured *Xanthobacteraceae* | -0.11 | -0.06 | -0.08 | 0.07 | -0.10 | -0.08 | 0.08 | 0.15 | 0.03 | 0.06 | -0.07 |
| *Microvirga* | -0.18 | -0.04 | 0.13 | -0.16 | 0.21 | 0.14 | 0.18 | -0.09 | 0.01 | 0.06 | -0.05 |
| bacteriap25 | -0.16 | -0.15 | -0.11 | 0.21 | 0.27 | -0.19 | -0.24 | 0.01 | -0.10 | -0.09 | -0.08 |
| uncultured bacterium *Elsterales* | -0.09 | -0.06 | -0.11 | 0.04 | -0.10 | -0.06 | 0.11 | 0.16 | 0.04 | 0.06 | -0.07 |
| *Bradyrhizobium* | -0.08 | 0.04 | -0.04 | 0.05 | -0.14 | -0.07 | 0.07 | 0.14 | 0.02 | 0.05 | -0.08 |
| *Pseudomonas* | **0.31** | 0.01 | **0.56** | -0.05 | -0.24 | 0.06 | -0.01 | -0.06 | 0.00 | 0.07 | 0.00 |
| *Streptomyces* | 0.18 | -0.19 | -0.13 | 0.11 | -0.08 | -0.06 | -0.13 | 0.13 | 0.00 | 0.03 | 0.06 |
| *Micromonosporaceae* | 0.20 | 0.01 | 0.03 | -0.12 | -0.14 | 0.03 | -0.09 | 0.09 | 0.07 | 0.03 | 0.16 |
| *Micromonospora* | 0.15 | -0.19 | -0.13 | -0.05 | 0.01 | 0.06 | 0.07 | 0.15 | 0.06 | 0.07 | 0.05 |
| uncultured bacterium *Gaiellales* | **0.32** | 0.08 | 0.06 | -0.08 | **-0.41** | -0.04 | -0.17 | -0.11 | -0.03 | -0.02 | 0.05 |
| *Nocardioides* | 0.15 | -0.07 | -0.21 | -0.27 | 0.25 | 0.19 | 0.29 | 0.05 | 0.11 | 0.09 | 0.14 |
| *Acidothermus* | 0.25 | 0.01 | 0.02 | 0.23 | **-0.58** | -0.23 | -0.17 | -0.02 | -0.14 | -0.02 | -0.15 |
| uncultured *Roseiflexaceae* | 0.08 | -0.08 | -0.05 | -0.02 | 0.28 | 0.05 | 0.01 | -0.03 | -0.01 | -0.07 | 0.03 |
| uncultured bacterium JG30-KF-AS9 | 0.22 | 0.00 | 0.11 | 0.01 | **-0.54** | -0.03 | -0.18 | 0.08 | 0.02 | 0.11 | 0.13 |
| Subgroup 6 | 0.02 | -0.18 | -0.03 | -0.26 | 0.25 | -0.03 | 0.08 | 0.03 | 0.13 | 0.05 | -0.06 |
| uncultured bacterium Subgroup 6 | -0.12 | -0.20 | -0.16 | -0.18 | **0.44** | -0.07 | 0.10 | 0.09 | 0.12 | 0.05 | -0.06 |
| uncultured bacterium Subgroup 2 | -0.09 | -0.08 | -0.11 | 0.08 | -0.10 | -0.08 | 0.10 | 0.17 | 0.03 | 0.06 | -0.07 |
| *Bacillus* | 0.22 | -0.10 | 0.30 | 0.11 | **-0.40** | -0.22 | -0.25 | 0.06 | -0.05 | 0.01 | -0.13 |
| *Dialister* | -0.19 | 0.00 | 0.05 | 0.14 | -0.14 | 0.01 | -0.09 | 0.07 | 0.02 | 0.17 | 0.10 |
| uncultured *Gemmatimonadaceae* | -0.05 | -0.16 | -0.08 | 0.08 | 0.22 | 0.02 | -0.06 | -0.12 | -0.08 | -0.06 | -0.02 |
| *Candidatus* Xiphinematobacter | -0.09 | -0.08 | -0.10 | 0.07 | -0.10 | -0.08 | 0.09 | 0.16 | 0.02 | 0.05 | -0.08 |
| uncultured *Nostocales* | 0.03 | 0.07 | -0.04 | -0.21 | 0.08 | 0.07 | -0.08 | 0.01 | 0.09 | -0.01 | 0.18 |
| *Nitrospira* | -0.05 | 0.12 | **0.54** | -0.03 | **-0.54** | 0.02 | -0.12 | 0.03 | -0.04 | 0.09 | -0.01 |

Values in bold indicate significant correlations (*p* < 0.05).
